# Beyond accuracy: evaluating the operational feasibility and diagnostic yield of CAD4TB vs. Timika score for scalable TB screening in low-resource settings

**DOI:** 10.3389/fdgth.2026.1748825

**Published:** 2026-05-29

**Authors:** Reyhan Eddy Yunus, Arierta Pujitresnani, Syarifaha Ihsan, Kahlil Gibran, Muhammad Reynalzi Yugo, Dean Handimulya Djumaryo, Prasandhya Astagiri Yusuf, Eric Daniel Tenda

**Affiliations:** 1 Medical Technology Cluster, Indonesian Medical Education and Research Institute, Faculty of Medicine, Universitas Indonesia, Jakarta, Indonesia; 2 Department of Radiology, Faculty of Medicine Universitas Indonesia, Dr. Cipto Mangunkusumo National General Hospital, Jakarta, Indonesia; 3 Department of Medical Physiology and Biophysics, Faculty of Medicine, Universitas Indonesia, Jakarta, Indonesia; 4 Department of Clinical Pathology, Faculty of Medicine Universitas Indonesia, Dr. Cipto Mangunkusumo National General Hospital, Jakarta, Indonesia; 5 Division of Respirology and Critical Illness, Department of Internal Medicine, Faculty of Medicine Universitas Indonesia, Dr. Cipto Mangunkusumo National General Hospital, Jakarta, Indonesia

**Keywords:** artificial intelligence, CAD4TB, chest x-ray, diagnosis, Timika scoring system, tuberculosis

## Abstract

Artificial intelligence has shown promise in enhancing tuberculosis care, but its use in resource-limited settings like Indonesia remains underexplored. This cross-sectional retrospective single-centre study evaluates the diagnostic performance of CAD4TB in screening Indonesian patients suspected of tuberculosis using chest x-ray (CXR) images, comparing its efficacy to the Timika score assessed by experts. We analyzed CXR images from 3,254 patients (2018–2020), including 600 with microbiological confirmation, of whom 46 had smear-positive pulmonary tuberculosis (PTB). CAD4TB demonstrated an area under the curve (AUC) of 0.778 (95% CI 0.712–0.844) when compared to acid-fast bacilli (AFB) results without a time interval. With a ≤7-day interval between CXR and AFB data, CAD4TB showed an AUC of 0.767 (95% CI 0.668–0.866), comparable to the Timika score of 0.726 (95% CI 0.632–0.820). Additionally, CAD4TB exhibited superior specificity (71.43% vs. 57.64%, *p* < 0.001) while maintaining a fixed sensitivity of 73.91%. These findings suggest that CAD4TB outperforms the Timika score and holds promise as a rapid tuberculosis screening tool in resource-limited settings like Indonesia.

## 1 Introduction

Tuberculosis, caused by the infection of Mycobacterium tuberculosis, constitutes a significant burden on public health, ranking among the top 10 causes of global mortality and representing the leading cause of death attributable to a single infectious agent (1). The prevalence of tuberculosis remains pronounced, with a substantial portion of cases concentrated in the Southeast Asian region, comprising 44% of the global burden in 2019. Notably, Indonesia emerges as one of the most affected countries, with an annual incidence rate of 845,000 tuberculosis cases, positioning it second worldwide after India in terms of tuberculosis burden (2). Despite the increasing tuberculosis case notification rate in Indonesia (2), misdiagnosis remains a significant challenge in the management of suspected tuberculosis patients (3). Notably, a previous study found that diagnostic errors occur in about 97.5% of tuberculosis patients (3). In Indonesia, although no misdiagnosis rate has been reported yet, only 56% of tuberculosis cases reported in 2021 were bacteriologically confirmed (4). The limited resources in Indonesia poses significant challenges, particularly in rural areas where bacteriological analysis and GeneXpert MTB/RIF are not widely available. As a result, rapid detection using chest x-ray (CXR) is essential. However, the availability of clinicians and radiologists to interpret the CXR is limited especially in most rural regions. This suggests the urgency to improve the diagnostic care of tuberculosis patients in Indonesia (1).

In recent years, artificial intelligence (AI) has demonstrated promising potential in improving tuberculosis care. By leveraging large datasets comprising clinical data, radiological imaging, and bacteriological testing, several AI implementation methods such as Computer Vision (5), Natural Language Processing (NLP) (6), Chatbots, and Virtual Health Assistants (7), as well as machine learning and deep learning models can effectively enhance diagnostics to predict tuberculosis, facilitating rapid and accurate identification of affected individuals (8). There are several CXR-based AI solutions available in the market such as Lunit INSIGHT CXR, CAD4TB, InferRead DR, and JF CXR-1 - in which CAD4TB and qXR being the two top performers (9). The credibility of CAD4TB for TB screening is further reinforced by its endorsement from the World Health Organization (WHO) through the Stop TB Partnership, which recognizes the potential of AI-based radiographic tools to support tuberculosis screening programs, particularly in resource-limited settings (10). Nonetheless, the integration of AI in tuberculosis care in developing countries, including Indonesia, faces challenges due to the scarcity of large and diverse datasets, particularly well-annotated and high-quality CXR images essential for training robust algorithms (11). These algorithms may identify subtle radiological patterns and abnormalities undetectable by human eyes (12).

Computer-Aided Detection “4” Tuberculosis (CAD4TB) is one such AI-driven software used for screening tuberculosis patients through CXR images. CAD4TB demonstrates superior diagnostic accuracy with high sensitivity and specificity and closely matches the performance of expert observers (13). It offers exceptional efficiency and cost-effectiveness for tuberculosis screening by providing higher throughput, lower costs (13), and serving as a viable alternative for TB prevalence surveys, especially in resource-limited settings (14). This preliminary investigation was conducted to assess the feasibility of implementing CAD4TB within the Indonesian population. Therefore, this study aims to measure and evaluate the diagnostic capability of CAD4TB in identifying tuberculosis patients in Indonesia using CXR images by comparing its accuracy to the Timika score and radiological reference, both assessed by experts.

## 2 Methods

### 2.1 Study design and patients

This cross-sectional retrospective single center study included adult patient data (above 18 years old) presenting with suspected tuberculosis and having available microbiological data at Dr Cipto Mangunkusumo National Referral Hospital, Jakarta, Indonesia, from 2018 to 2020. Only initial evaluations were included, and no follow-up data were analyzed. Clinical data and CXR images were retrieved from medical records and the Picture Archiving and Communication System (PACS). Pregnant women and patients without confirmed microbiological findings or with poor CXR image quality were excluded from the study.

The case definition in this study follows the Indonesian National Guidelines for Medical Services (Pedoman Nasional Pelayanan Kedokteran, PNPK) for Tuberculosis Management (15). In accordance with these guidelines, pulmonary TB diagnosis is primarily established based on microbiological evidence (smear microscopy, molecular testing/TCM, or culture), while clinical findings (PTB symptoms) and radiological findings (CXR interpretation) serve as supportive information. Chest x-ray cannot be used as a sole diagnostic basis due to its non-specific nature. Accordingly, PTB cases in this study were defined based on microbiological results, distinguishing patients with AFB-positive findings and those without bacteriological confirmation, while still considering clinical and radiological findings as part of the overall assessment.

AFB results were used as the gold standard to measure and evaluate the diagnostic performance of CAD4TB and Timika score. AFB testing detects the presence of acid-fast bacilli (Mycobacterium spp.) in patients with suspected TB and is not specific for Mycobacterium tuberculosis (MTB). However, as Indonesia is a TB-endemic country, AFB smear remains the most accessible and cost-effective initial diagnostic method.

The interval between imaging and microbiological testing can influence diagnostic accuracy, affecting both sensitivity and specificity. To ensure consistency with clinical guidelines, a seven-day cut-off between CXR and microbiological examination was applied (15). A subset analysis was then performed on patients within this interval to compare the diagnostic performance of CAD4TB with expert radiological interpretations.

### 2.2 Artificial intelligence algorithm for chest x-ray interpretation

We utilized the offline CAD4TBbox version 7 software (Delft Imaging, Hertogenbosch, The Netherlands) to automate CXR interpretation for the selected patients. CAD4TB employs trained deep learning algorithms and deep neural networks to automatically interpret chest radiographs for signs of tuberculosis (16, 17). In brief, the program analyzes CXR images for tuberculosis patterns using the color heat-map method through the following steps (18):

1. Digital CXR from selected patients in DICOM format was uploaded to local storage “CAD4TBbox” to ensure data remains within the country and enables usage in rural areas without or with unreliable internet connection.
2. CXR scoring was done automatically in the software in six consecutive steps:
  2.1. Normalization: to normalize the size of the CXR images for the AI algorithm to process the images uniformly.
  2.2. Lung field segmentation: to delineate the lungs and distinguish them from the other structures.
  2.3. Texture analysis: to determine relevant abnormalities in lung segments.
  2.4. Area analysis: to estimate the percentage of abnormal lung parenchyma.
  2.5. All filter weights were calculated. The average filter weight was used as a mask on the CXR image to generate a color heat map, which was visualized only on the lung DIFFERarea segmented by the previously trained model.
  2.6. The color heat map produced different colors corresponding to its weight, denoting the probability of present pathology on the CXR as follows: red (high), yellow (medium), green (low), and blue (very low).

The digital CXR was input on CAD4TB software to generate two AI scorings: 1) the affected lung area score (range 0–100), defined as the percentage of detected abnormalities in the lung parenchyma relative to the total lung volume, and 2) the tuberculosis score (range 0–100), determined by the average final weight of all layers.

### 2.3 Timika scoring system and radiological reference

The CXR images of the included patients were independently assessed by two radiologists (MRY and WS) with five and ten years of experience, respectively. The two raters evaluated the posteroanterior CXR images using the Timika scoring system (range 0–140), a grading tool to assess the severity of tuberculosis (19), in a blinded fashion where the raters were unaware of each other’s total scoring, AI scoring results, and clinical and microbiological findings. The details of the scoring system are outlined in Table 1. Mean Timika score from the two radiologists was later computed for the diagnostic analysis.

**Table 1:** Calculation of the timika score (18).

Calculation of the Timika Score from CXR
Posteroanterior chest x-ray (CXR-PA) images with adequate positioning and penetration were used. Each image was divided into six lung zones using two horizontal reference lines.
For each zone, the estimated proportion of lung involvement (e.g., consolidation or nodules) was visually assessed.
The percentages from all six zones were summed and normalized to obtain the overall percentage of lung involvement.
If cavitation was present on the CXR, an additional value of 40 was added to the score.
Final CXR score=percentage of lung involvement+40 (if cavitation present).

In addition to the Timika scoring, the raters also classified the CXR images into four categorical groups as radiological reference, which were subsequently dichotomized into positive (suspected active tuberculosis) and negative (inactive, indeterminate, or non-tuberculosis) groups for diagnostic performance assessments (20).

### 2.4 Data analysis

Descriptive data were presented as frequency (n) and proportion (%) in this study. Diagnostic parameters, including sensitivity, specificity, positive predictive value (PPV), negative predictive value (NPV), receiver operating characteristic (ROC) curve, and the area under curve (AUC), were estimated to compare the accuracy of CAD4TB and the expert-assessed Timika score (each rater individually and the mean from the two raters) against AFB staining results as the gold standard. Bootstrapping techniques were employed to estimate the 95% CIs of the ROC curve. Cut-off sensitivities of >70%, >80%, and >90% were utilized to assess the specificity, PPV, NPV, and AUC of the CAD4TB and the Timika score. Additionally, the specificity between CAD4TB and Timika score was compared using McNemar tests, with statistical significance set at 5%. We also calculated the AUC for CAD4TB compared to the Dichotomous Group categorization by the raters as a gold reference standard to evaluate the comparison of accuracy with results against AFB.

## 3 Results

### 3.1 Patients characteristics and sample output of AI system

**Figure 1:**
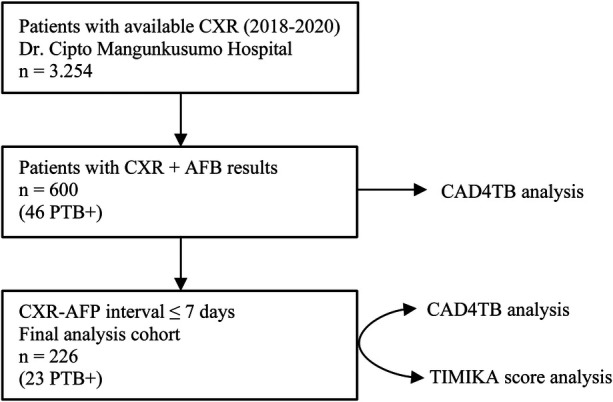
Flowchart of patient selection and dataset refinement.

**Figure 2:**
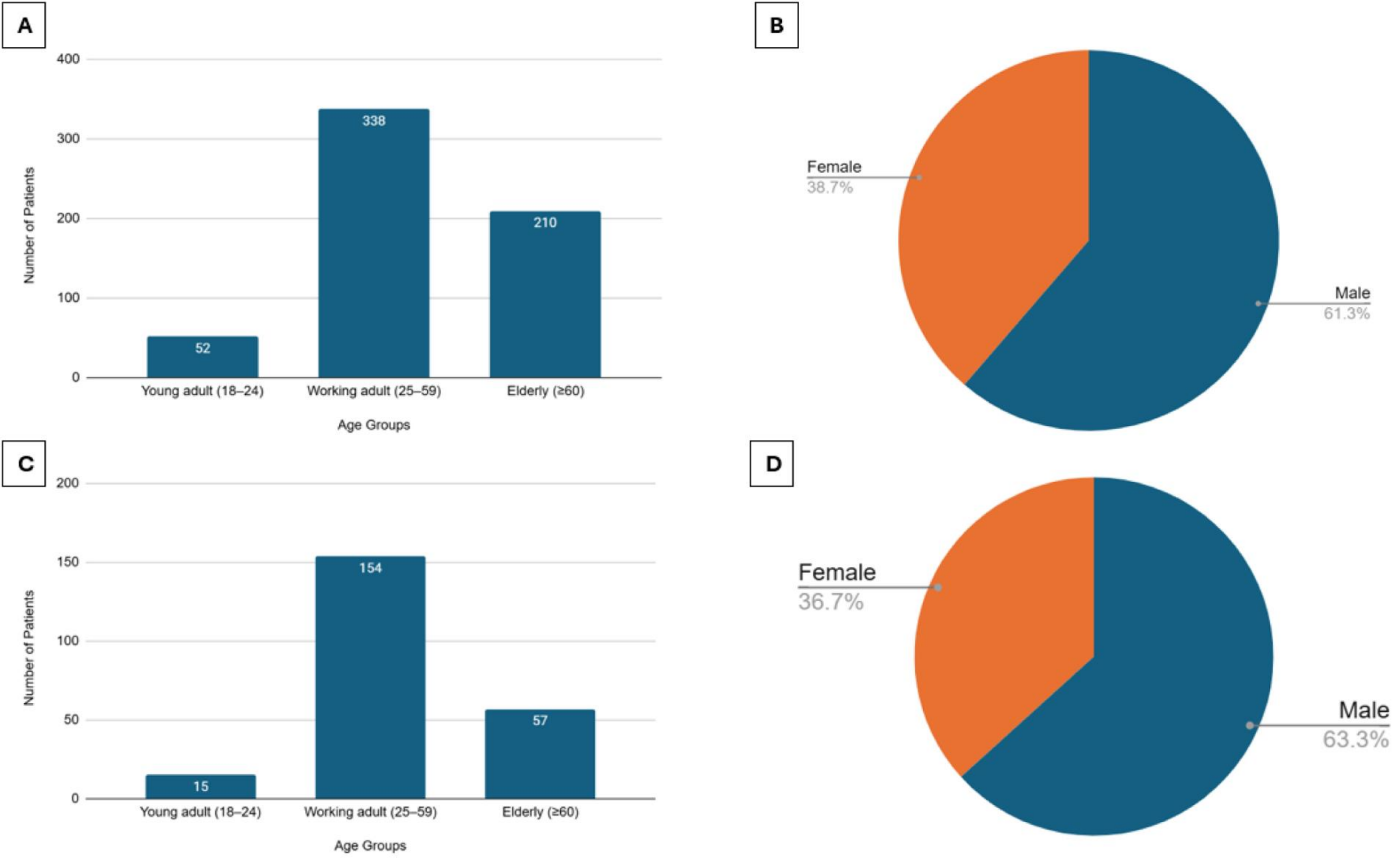
Distribution of patients by age group and sex. **(A, B)** Full cohort (*n* = 600); **(C, D)** final analysis subset (*n* = 226). **(A, C)** Age group distribution; **(B, D)** sex distribution.

**Figure 3:**
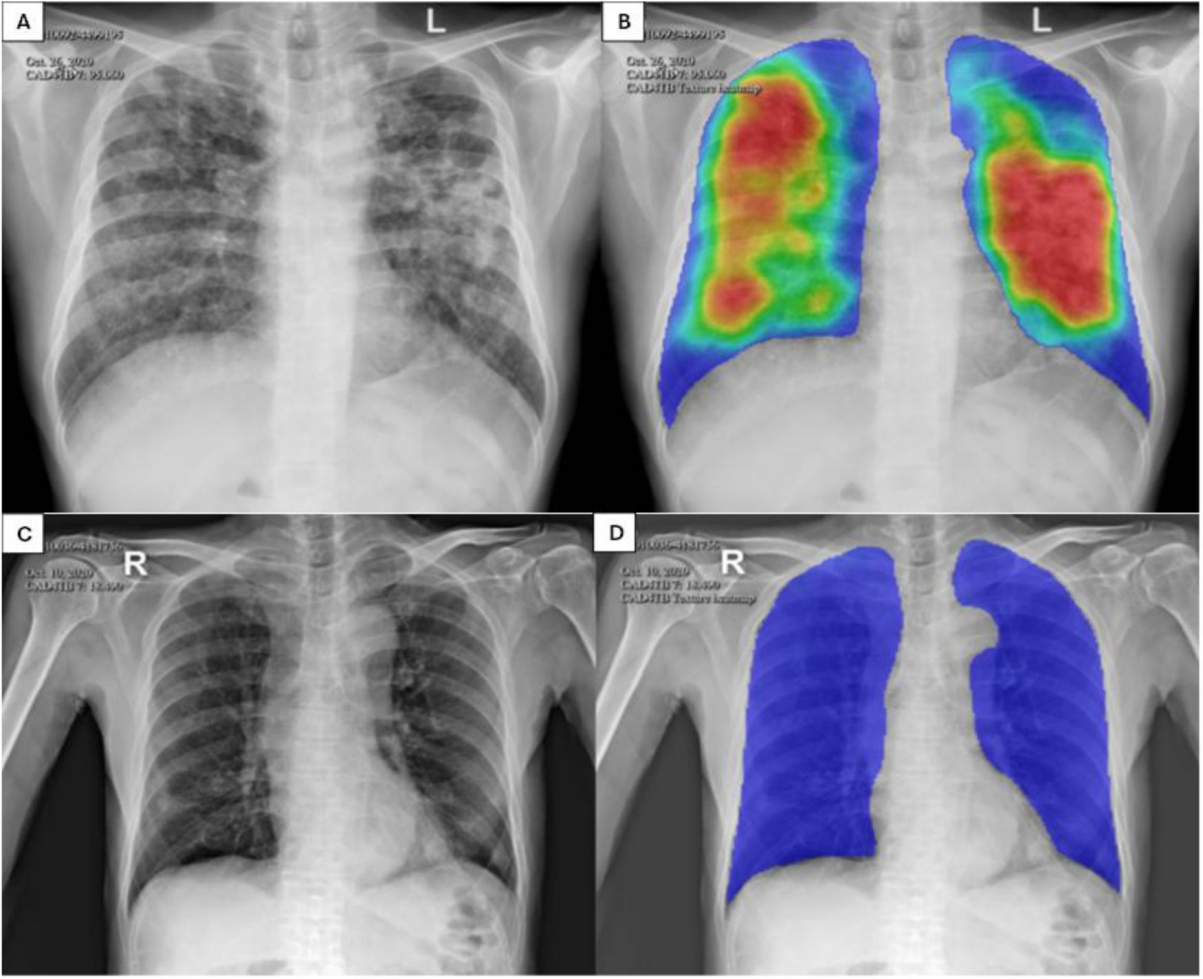
**(A)** example of original chest x-ray image of a positive tuberculosis patient confirmed by AFB smear. **(B)** The output of CAD4TB analysis from **(A)** with a score of 95.06. **(C)** Example of original chest x-ray image of a negative tuberculosis patient confirmed by AFB smear. **(D)** The output of CAD4TB analysis from **(C)** with a score of 18.49.

Out of 3,254 patients with available CXR images, only 600 (18.44%) had AFB results (Table 2). The mean age of patients was 49.5 ± 16.1 years, ranging from 19 to 86 years. Based on age grouping according to the Indonesian Ministry of Health classification, the majority of patients were in the working adult group (25–59 years), followed by elderly (≥60 years) and young adults (18–24 years), as illustrated in Figure 2A. Based on AFB smear results, 46 patients (7.18%) were classified as PTB-positive, with a predominance of male patients (61.3%) (Figure 2B). Of the 600 patients, 226 (36.04%) had a CXR–AFB interval of ≤7 days and were included in the final analysis (Table 2; Figure 1). The age and sex distribution of this subset remained comparable to the full cohort, with working adults still representing the largest proportion, followed by elderly and young adults, and a predominance of male patients (Figures 2C, D). Although derived from a subset of cases in a single center, the data were collected from Indonesia’s national referral hospital, which serves a demographically diverse population. Figure 3 presents representative CAD4TB outputs for patients with positive and negative AFB results.

**Table 2:** Number of patients screened for inclusion from 2018 to 2020.

Year	*N* of patients with available CXR	*N* of patients with available CXR and AFB results	*N* of patients with available CXR and AFB results with an interval of ≤7 days
2018	1,327	96 (9 PTB+)	53 (7 PTB+)
2019	1,083	322 (18 PTB+)	92 (4 PTB+)
2020	844	182 (19 PTB+)	81 (12 PTB+)
Total	**3,254**	**600 (46 PTB+)**	**226 (23 PTB+)**

AFB, acid-fast bacilli; CXR, chest x-ray; PTB, pulmonary tuberculosis. Bold values indicate the total sum.

### 3.2 Diagnostic accuracy of CAD4TB and Timika score in detecting PTB

**Figure 4:**
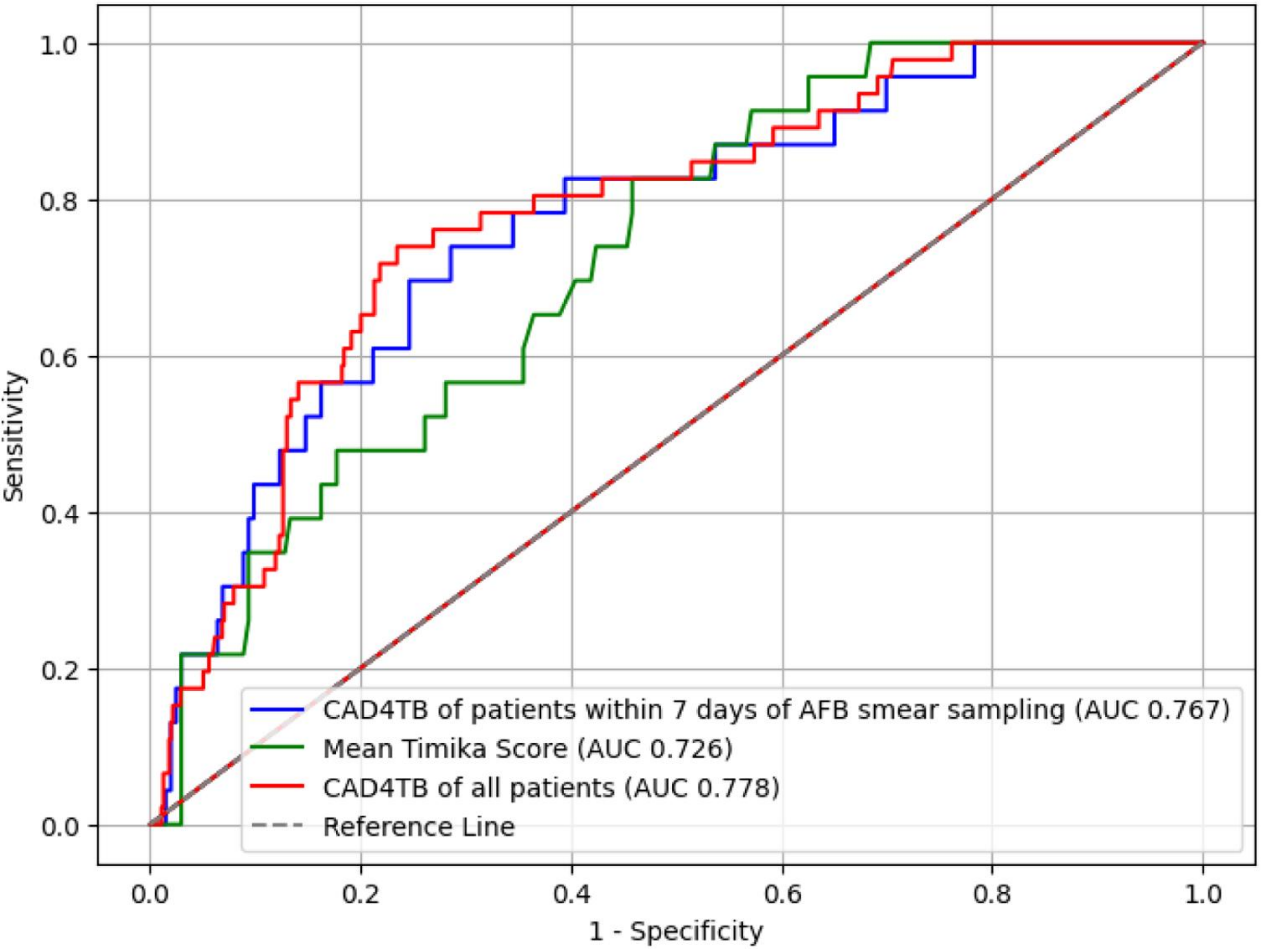
ROC curve illustrates the diagnostic performance of CAD4TB without time restrictions on AFB smear sampling (red line) and CAD4TB within a maximum range of 7 days after AFB smear sampling (blue line), as well as its comparison with the Timika score (green line) in detecting PTB using AFB as the reference standard, based on CXR data. AUC, area under the curve; CAD4TB, Computer-Aided Detection “4” Tuberculosis.

Using AFB as the reference standard, the diagnostic accuracy of CAD4TB yielded an AUC of 0.778 [95% confidence interval (CI) 0.712–0.844], slightly higher compared to the diagnostic accuracy of CAD4TB of patients within a maximum range of 7 days of AFB smear sampling with AUC of 0.767 [0.668–0.866]. This result was comparable to the mean Timika score [0.726 (0.632–0.820)] (Figure 4). We determined the cut-off value to categorize the estimated PTB based on the CAD4TB score, yielding N PTB+ and N PTB-, as shown in Table 3. The specificity of CAD4TB in detecting PTB was higher than that of the Timika score when sensitivity was set at 73.91% (71.43% vs. 57.64%, *p* < 0.001) and 82.61% (60.59% vs. 54.19%, *p*=0.112). However, when sensitivity increased to 91.3%, the specificity of CAD4TB became lower than that of the Timika score (34.98% vs. 42.86%, *p*=0.053). Further details are presented in the table below.

**Table 3:** Diagnostic parameters of CAD4TB and the Timika score under different fixed sensitivity settings.

Diagnostic modality	Sensitivity	Timika		CAD4TB Specificity	*P* ^a^	PPV	NPV	N PTB+	N PTB-	AUC (95%CI)
		Cut-off	Specificity							
All Data										
CAD4TB (>70%)	71.74%			78.16%		21.43%	97.30%	154	446	0.778 (0.712–0.844)
CAD4TB (>80%)	80.43%			63.54%		15.48%	97.51%	239	361	0.778 (0.712–0.844)
CAD4TB (>90%)	91.30%			36.46%		10.66%	98.06%	394	206	0.778 (0.712–0.844)
Sensitivity score 1 (>70%)										
CAD4TB	73.91%			71.43%		21.62%	95.39%	74	152	0.767 (0.668–0.866)
Mean Timika	73.90%	52.71	57.64%		0.053	16.50%	95.12%	103	123	0.726 (0.632–0.820)
Sensitivity score 2 (>80%)										
CAD4TB	82.61%			60.59%		19.19%	96.85%	99	127	0.767 (0.668–0.866)
Mean Timika	82.61%	45.21	54.19%		0.053	16.96%	96.49%	112	114	0.726 (0.632–0.820)
Sensitivity score 3 (>90%)										
CAD4TB	91.30%			34.98%		13.73%	97.26%	153	73	0.767 (0.668–0.866)
Mean Timika	91.30%	23.34	42.86%		0.053	15.33%	97.75%	137	89	0.726 (0.632–0.820)

AUC, area under the curve; CAD4TB, Computer-Aided Detection “4” Tuberculosis; CI, confidence interval; PPV, positive predictive value; NPV, negative predictive value; N PTB+ and N PTB-, estimated positive and negative pulmonary tuberculosis based on certain CAD4TB score cut-off.

a *P*-values derived from McNemar tests, comparing the specificity between CAD4TB and Timika score.

Subgroup analysis indicated that both the sensitivity and specificity of the Timika score were slightly higher in 2019 compared to 2018 and 2020 across all sensitivity values (Supplementary Table S1). Despite these variations, the AUC of the mean Timika score remained consistent throughout the study period, with values of 0.677 [95%CI 0.484–0.870] in 2018, 0.743 [0.561–0.913] in 2019, and 0.719 [0.567–0.871] in 2020 (Supplementary Table S2).

In contrast, the AUC of CAD4TB was slightly lower in 2020 [0.682 (0.515–0.849)] compared to 2018 [0.839 (0.721–0.956)] and 2019 [0.821 (0.617–1.000)] (Supplementary Table S2). However, the specificity of CAD4TB remained stable across the study period (Supplementary Table S3), suggesting that while its sensitivity fluctuated, the model’s ability to identify non-TB cases correctly was reliable. When directly compared to the Timika score, CAD4TB demonstrated comparable specificity across the entire study period from 2018 to 2020 (Supplementary Tables S4, S5 and Supplementary Figure S1), reinforcing its capability as an effective diagnostic tool despite temporal variability in AUC values.

### 3.3 Radiological reference as the ground truth for CAD4TB diagnostic test

**Figure 5:**
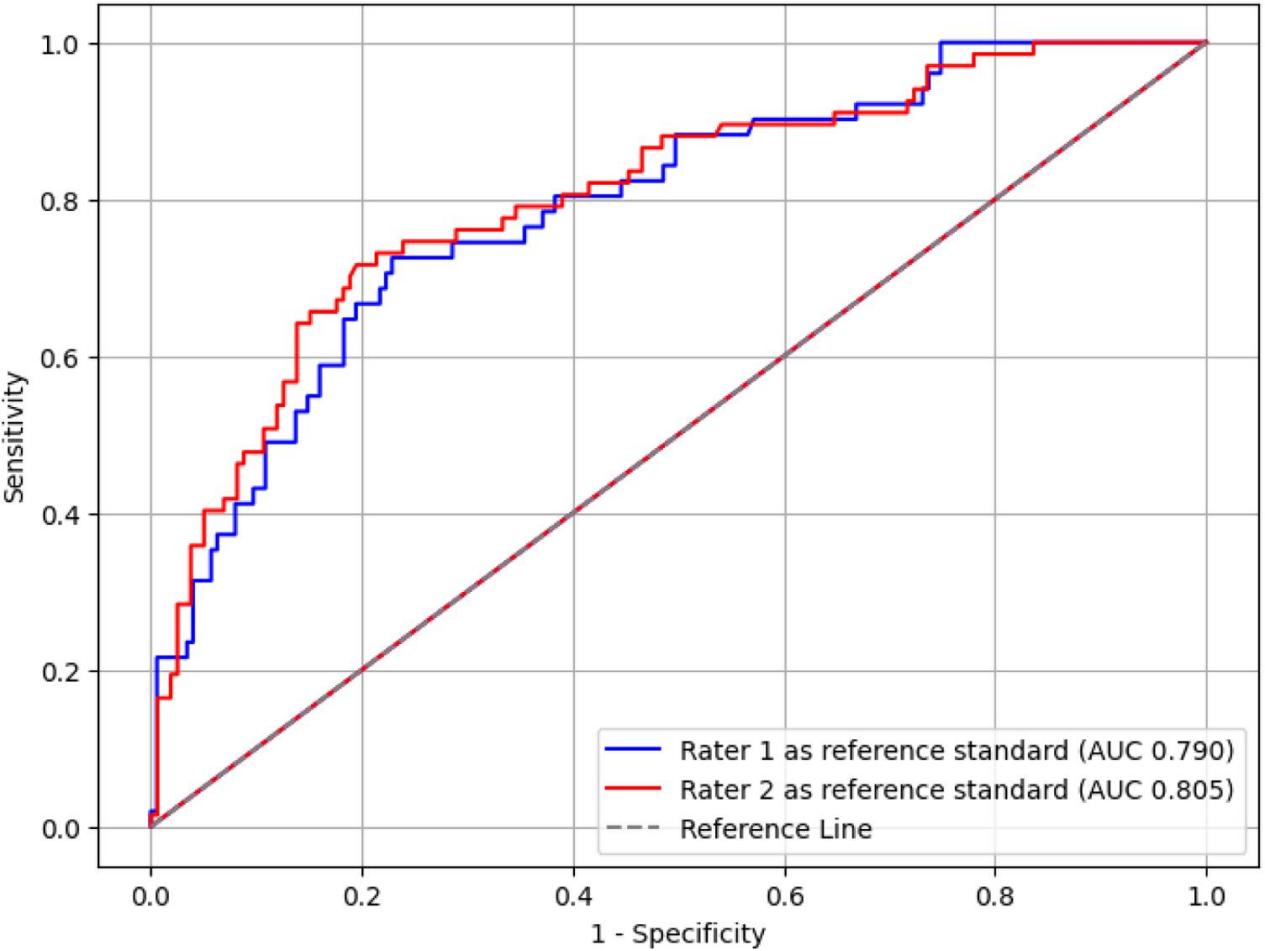
ROC curve showing the diagnostic ability of CAD4TB in detecting PTB using the radiological assessments from rater 1 (blue line) and rater 2 (red line) in the dichotomous group as a reference standard. AUC, the area under the curve.

Although AFB is the gold standard to determine positive tuberculosis, clinical evidence from CXRs is often used by pulmonologists and radiologists for faster and more reliable results in resource-limited settings. Here, we subsequently analyze the diagnostic test of CAD4TB using radiological reference as the ground truth - since both scores solely use only CXR to diagnose TB. The AUC values obtained using this analysis were 0.790 [0.720–0.861] using rater-1 as reference standard and 0.805 [0.741–0.868] using rater-2 as reference standard (Figure 5).

### 3.4 Interrater reliability of the Timika scoring system and radiological reference in classifying PTB

**Figure 6:**
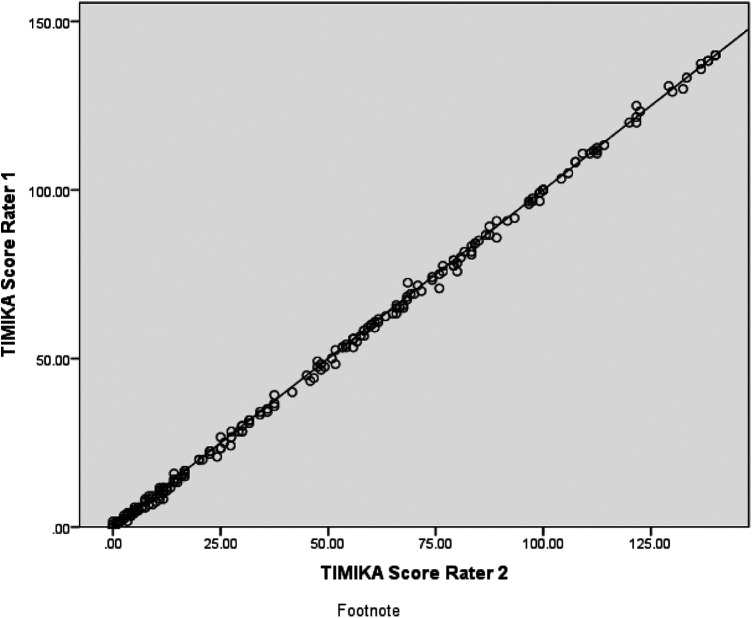
Interrater agreement in assessing the Timika score.

The Timika score between the first (MRY) and the second (WS) raters showed a highly positive correlation (*ρ*=0.999, *p* < 0.001; Figure 6). As for the radiological reference, the assessment showed a high level of reliability between the two radiologists in classifying the CXR reading results into dichotomous groups: positive (suspected active tuberculosis) and negative (inactive, indeterminate, or non-tuberculosis) (*κ* = 0.806 ± 0.032, 95% CI 0.743–0.869, *p* <0.05).

## 4 Discussion

The present study demonstrates that CAD4TB v7 shows good diagnostic performance when applied to a substantial dataset of CXR images from patients suspected of tuberculosis in Jakarta, Indonesia (21, 22). In our clinical setting, CAD4TB achieved diagnostic accuracy comparable to the Timika score assessed by two expert radiologists, despite differences in their levels of experience. This finding highlights the potential of CAD4TB to provide faster, more objective, and standardized CXR interpretation, which is particularly valuable in settings with limited radiological expertise.

Our findings are consistent with previous studies, showing comparable performance to CAD4TB v6 [AUC 0.83 (95% CI 0.82–0.84)] and improved performance compared to earlier versions such as CAD4TB v3.07 (AUC 0.423) (23, 24). Furthermore, consistent with prior reports, CAD4TB demonstrated slightly lower sensitivity in detecting PTB among smear-negative patients, indicating that further algorithm refinement is needed, particularly for cases with atypical presentations or higher likelihood of false-negative microbiological results, such as in patients with HIV infection or asymptomatic disease (25).

To further contextualize these findings, we evaluated CAD4TB and the Timika score across three fixed sensitivity thresholds (>70%, >80%, and >90%), representing different clinical use scenarios. As expected, increasing the sensitivity threshold results in reduced specificity, which explains why CAD4TB may exhibit lower specificity than the Timika score at higher sensitivity settings. In high-burden settings such as Indonesia, higher sensitivity thresholds are preferred for screening to minimize missed TB cases, albeit at the expense of more false positives. Conversely, lower thresholds may be more appropriate for triage or confirmatory settings, where improved specificity helps reduce unnecessary follow-up testing. Across these thresholds, CAD4TB demonstrated performance comparable to the Timika score, while offering advantages in terms of rapid, standardized, and operator-independent interpretation.

Notably, CAD4TB can interpret CXR images in approximately 20 s, facilitating prompt diagnosis and treatment of suspected patients (22). However, it is important to emphasize that CAD4TB does not replace microbiological confirmation, particularly using Xpert MTB/RIF, which remains the first-line diagnostic test for suspected tuberculosis cases. In situations where microbiological results are indeterminate or access to microbiological tests is limited, CAD4TB may serve as a prioritized diagnostic tool (22). Nonetheless, consistent with previous studies by Tavaziva et al., CAD4TB version 6 showed slightly lower sensitivity in detecting PTB in smear-negative patients [−12.3% (95%CI −19.5, −6.1%)], which aligns with our findings (23). This indicates that further development of CAD4TB algorithms is still required to enhance its diagnostic accuracy in patients with a higher likelihood of false-negative microbiological findings such as patients with human immunodeficiency virus infection and asymptomatic disease (25).

This study offers significant value as it represents the first to directly compare the diagnostic accuracy of CAD4TB to the expert-assessed Timika score. Employing concurrent and blinded assessments of the same CXR dataset allowed for a robust and direct comparison between AI-driven detection and human interpretations, highlighting the potential of this AI software in clinical practice. Furthermore, the use of data from a national referral hospital provides a diverse and clinically relevant patient population, enhancing the applicability of the findings in real-world settings.

Despite these strengths, several limitations should be acknowledged. The present study was limited by the relatively with imbalance data between confirmed positive and negative TB used in the evaluation of the CAD4TB system, which may pose risks of both overfitting and underfitting. It is important to note that the CAD4TB system was pre-trained and not developed or trained as part of this study, and its performance may depend on the quality and representativeness of training data, as well as the need for periodic updates and calibration (26).

The study was conducted at a tertiary referral hospital where patients tend to have more severe disease or atypical presentations, potentially limiting generalizability to primary care settings. Consequently, this may limit its applicability to general tuberculosis patients who usually seek care at primary health facilities. Moreover, the limited availability of clinical and laboratory data might have affected the precision of the system, although it likely had minimal influence on the study results. In addition, the number of PTB-positive cases was relatively low, which may introduce a wider margin of error; however, this reflects real-world screening conditions with low PTB prevalence, and robustness was maintained through appropriate statistical methods (ROC and bootstrapping).

Additionally, our study was affected by the COVID-19 pandemic, during which the number of tuberculosis patients presenting to our center decreased remarkably, while the number of COVID-19 patients presenting with pulmonary complications increased. These shifts in patient characteristics, evidenced by the lower AUC of CAD4TB in 2020 compared to previous years (Supplementary Table S2), imply that a higher proportion of non-tuberculosis patients may have exhibited radiographic patterns similar to tuberculosis. Overlap in chest x-ray findings between TB and COVID-19 may contribute to misclassification and reduced diagnostic performance, as supported by previous studies (27, 28).

Further research with larger datasets integrating CXR images with clinical signs and symptoms and laboratory data is needed to better evaluate the diagnostic performance of CAD4TB in detecting PTB. In addition, external validation is essential to assess the generalizability of the model; however, as this study was conducted in a single-center setting, such validation was beyond the scope of the current work and will be addressed in future studies using multi-center datasets. Additionally, insights into the cost-effectiveness, operational aspects, and integration of CAD4TB into tuberculosis management in Indonesia are essential for its effective implementation.

In conclusion, AI-driven CAD4TB demonstrated comparable diagnostic efficacy to the human-assessed Timika score in detecting PTB through CXR images. Its rapid and relatively accurate triage capability may enhance tuberculosis care in low-resource settings, such as Indonesia. However, future studies with larger and multicenter datasets, integrating clinical and radiological indices, are warranted to confirm these findings.

## Data Availability

The raw data supporting the conclusions of this article will be made available by the authors, without undue reservation.
