# Supplementary material for: Beyond accuracy: evaluating the operational feasibility and diagnostic yield of CAD4TB vs. Timika score for scalable TB screening in low-resource settings
Source: Front Digit Health. 2026 May 29;8:1748825. doi: 10.3389/fdgth.2026.1748825 (PMC13260493; doi:10.3389/fdgth.2026.1748825)

Supplementary Material

# Supplementary Table S1. Cut-off values and specificity of Timika scores compared to reference standard across varying fixed sensitivity values between 2018 and 2020.

|  | **Sensitivity** $\boldsymbol{\approx}$ **Specificity** | | | **Sensitivity >70%** | | | **Sensitivity >80%** | | | **Sensitivity >90%** | | |
| --- | --- | --- | --- | --- | --- | --- | --- | --- | --- | --- | --- | --- |
|  | **Timika 1** | **Timika 2** | **Mean Timika** | **Timika 1** | **Timika 2** | **Mean Timika** | **Timika 1** | **Timika 2** | **Mean Timika** | **Timika 1** | **Timika 2** | **Mean Timika** |
| **2018 (n=53)** | | | | | | | | | | | | |
| Cut-off | 60.42 | 60.42 | 60.42 | 59.17 | 59.17 | 59.17 | 39.59 | 39.59 | 39.59 | 20 | 20.84 | 20.42 |
| Sensitivity | 57.14% | 57.14% | 57.14% | 71.43% | 71.43% | 71.43% | 85.71% | 85.71% | 85.71% | 100% | 100% | 100% |
| Specificity | 54.35% | 54.35% | 54.35% | 52.17% | 52.17% | 52.17% | 45.65% | 45.65% | 45.65% | 39.13% | 39.13% | 39.13% |
| **2019 (n=92)** | | | | | | | | | | | | |
| Cut-off | 50.83 | 52.5 | 51.67 | 50.83 | 52.5 | 51.67 | 27.5 | 29.59 | 28.96 | 27.5 | 29.59 | 28.96 |
| Sensitivity | 75% | 75% | 75% | 75% | 75% | 75% | 100% | 100% | 100% | 100% | 100% | 100% |
| Specificity | 63.64% | 63.64% | 63.64% | 63.64% | 63.64% | 63.64% | 50% | 52.27% | 52.27% | 50% | 52.27% | 52.27% |
| **2020 (n=81)** | | | | | | | | | | | | |
| Cut-off | 66.25 | 66.25 | 65.63 | 55.42 | 55 | 54.79 | 43.75 | 46.25 | 45 | 14.59 | 15.84 | 15.2 |
| Sensitivity | 66.67% | 66.67% | 66.67% | 75% | 75% | 75% | 83.33% | 83.33% | 83.33% | 91.67% | 91.67% | 91.67% |
| Specificity | 68.12% | 66.67% | 66.67% | 59.42% | 57.97% | 57.97% | 50.72% | 50.72% | 50.72% | 31.88% | 31.88% | 31.88% |
| **2018-2020 (n=226)** | | | | | | | | | | | | |
| Cut-off | 59.59 | 59.59 | 59.59 | 52.91 | 52.5 | 52.7 | 43.75 | 46.25 | 45.21 | 22.92 | 24.59 | 23.34 |
| Sensitivity | 65.22% | 65.22% | 65.22% | 73.91% | 73.91% | 73.91% | 82.61% | 82.61% | 82.61% | 91.3% | 91.3% | 91.3% |
| Specificity | 64.04% | 63.55% | 63.55% | 57.64% | 57.64% | 57.64% | 53.69% | 54.19% | 54.19% | 42.86% | 42.86% | 42.86% |

# Supplementary Table S2. Area under the curve (AUC) values of CAD4TB and the Timika scores compared to between 2018 and 2020.

| **Comparison**  **(vs. Reference standard)** | **2018 (n=53)** | **2019 (n=92)** | **2020 (n=81)** | **2018-2020 (n=226)** |
| --- | --- | --- | --- | --- |
| Timika 1 | 0.677 (0.484-0.870) | 0.736 (0.558-0.914) | 0.719 (0.566-0.871) | 0.726 (0.631-0.820) |
| Timika 2 | 0.679 (0.484-0.873) | 0.745 (0.567-0.910) | 0.720 (0.569-0.872) | 0.727 (0.633-0.821) |
| Mean Timika | 0.677 (0.484-0.870) | 0.743 (0.561-0.913) | 0.719 (0.567-0.871) | 0.726 (0.632-0.820) |
| CAD4TB | 0.839 (0.721-0.956) | 0.821 (0.617-1.000) | 0.682 (0.515-0.849) | 0.767 (0.668-0.866) |

**Supplementary Table S3.** Cut-off values and specificity of CAD4TB compared to reference standard across varying fixed sensitivity values between 2018 and 2020.

|  | **2018 (n=53)** | | | | **2019 (n=92)** | | | | **2020 (n=81)** | | | | **2018-2020 (n=226)** | | | |
| --- | --- | --- | --- | --- | --- | --- | --- | --- | --- | --- | --- | --- | --- | --- | --- | --- |
|  | $\boldsymbol{\approx}$**Sp** | **>70%** | **>80%** | **>90%** | $\boldsymbol{\approx}$**Sp** | **>70%** | **>80%** | **>90%** | $\boldsymbol{\approx}$**Sp** | **>70%** | **>80%** | **>90%** | $\boldsymbol{\approx}$**Sp** | **>70%** | **>80%** | **>90%** |
| Cut-off | 74.09 | 74.09 | 73.93 | 70.42 | 71.88 | 84.13 | 61.82 | 61.82 | 71.85 | 69.06 | 55.07 | 51.39 | 72.25 | 72.03 | 69.06 | 55.07 |
| Sensitivity | 71.4% | 71.4% | 85.7% | 100% | 75% | 75% | 100% | 100% | 66.67% | 75% | 83.33% | 91.67% | 69.57% | 73.91% | 82.61% | 91.30% |
| Specificity | 76.10% | 76.10% | 76.10% | 67.40% | 75.00% | 89.77% | 47.73% | 47.73% | 66.67% | 59.42% | 30.43% | 24.64% | 71.43% | 71.43% | 60.59% | 34.98% |

**Supplementary Table S4.** Cut-off values and specificity of CAD4TB compared to Timika score from the first rater (RY) across varying fixed sensitivity values between 2018 and 2020.

|  | **2018 (n=53)** | | | | **2019 (n=92)** | | | | **2020 (n=81)** | | | | **2018-2020 (n=226)** | | | |
| --- | --- | --- | --- | --- | --- | --- | --- | --- | --- | --- | --- | --- | --- | --- | --- | --- |
|  | $\boldsymbol{\approx}$**Sp** | **>70%** | **>80%** | **>90%** | $\boldsymbol{\approx}$**Sp** | **>70%** | **>80%** | **>90%** | $\boldsymbol{\approx}$**Sp** | **>70%** | **>80%** | **>90%** | $\boldsymbol{\approx}$**Sp** | **>70%** | **>80%** | **>90%** |
| Cut-off | 70.42 | 70.42 | 68.78 | 61.235 | 69.68 | 65.515 | 61.25 | 49.455 | 72.315 | 73.98 | 71.845 | 60.63 | 71.255 | 72.025 | 67.85 | 56.625 |
| Sensitivity | 73.33% | 73.33% | 80.00% | 93.33% | 66.67% | 73.33% | 80.00% | 93.33% | 76.19% | 71.43% | 80.95% | 90.48% | 72.55% | 70.59% | 80.39% | 90.20% |
| Specificity | 71.05% | 71.05% | 65.79% | 57.89% | 66.23% | 54.55% | 48.05% | 28.57% | 76.67% | 78.33% | 76.67% | 48.33% | 72.57% | 77.71% | 61.71% | 42.86% |

**Supplementary Table S5.** Cut-off values and specificity of CAD4TB compared to Timika score from the second rater (WS) across varying fixed sensitivity values between 2018 and 2020.

|  | **2018 (n=53)** | | | | **2019 (n=92)** | | | | **2020 (n=81)** | | | | **2018-2020 (n=226)** | | | |
| --- | --- | --- | --- | --- | --- | --- | --- | --- | --- | --- | --- | --- | --- | --- | --- | --- |
|  | $\boldsymbol{\approx}$**Sp** | **>70%** | **>80%** | **>90%** | $\boldsymbol{\approx}$**Sp** | **>70%** | **>80%** | **>90%** | $\boldsymbol{\approx}$**Sp** | **>70%** | **>80%** | **>90%** | $\boldsymbol{\approx}$**Sp** | **>70%** | **>80%** | **>90%** |
| Cut-off | 71.295 | 74.085 | 70.42 | 61.235 | 69.52 | 69.52 | 56.56 | 48.615 | 71.255 | 72.315 | 67.74 | 58.795 | 70.715 | 71.75 | 66.27 | 51.695 |
| Sensitivity | 76.47% | 70.59% | 82.35% | 94.12% | 70.83% | 70.83% | 83.33% | 91.67% | 76.92% | 73.08% | 80.77% | 92.31% | 74.63% | 70.15% | 80.60% | 91.04% |
| Specificity | 77.78% | 88.89% | 77.78% | 61.11% | 70.59% | 70.59% | 42.65% | 27.94% | 76.36% | 80.00% | 63.64% | 49.09% | 74.84% | 81.13% | 61.01% | 35.22% |

**Supplementary Figure S1**. Receiver operating characteristic (ROC) curve showing the diagnostic ability of CAD4TB and Timika scores in detecting pulmonary tuberculosis in (a) 2018, (b) 2019, and (c) 2020


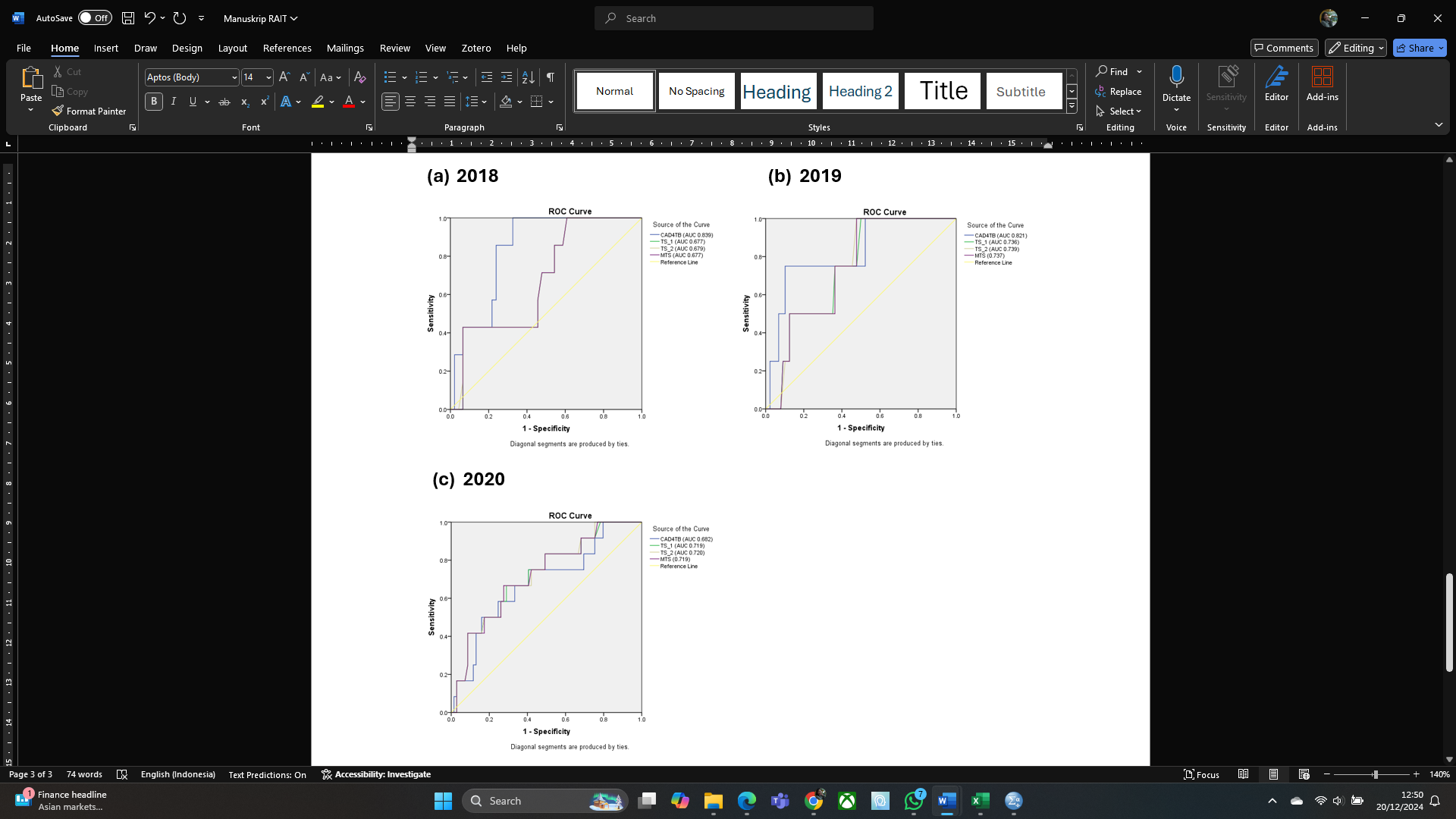

Supplement: Supplementary file 1 [file Table1.docx]
